# Supplementary figures and images for: The Effect of Radial-Shear Rolling Deformation Processing on the Structure and Properties of Zr-2.5Nb Alloy
Source: Materials (Basel). 2023 May 21;16(10):3873. doi: 10.3390/ma16103873 (PMC10222539; doi:10.3390/ma16103873)

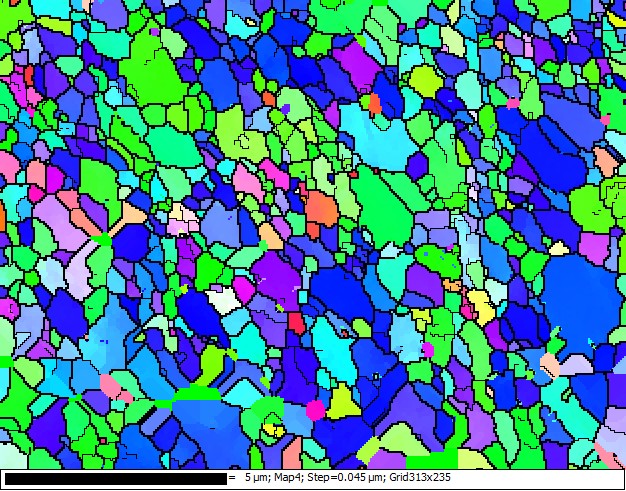

Supplement: Supplementary file 1 [file materials-16-03873-s001.zip › EBSD 0 mm distance from the center.jpg]

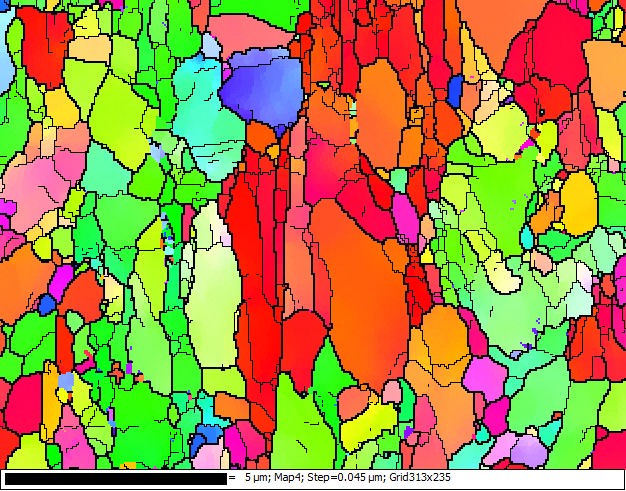

Supplement: Supplementary file 1 [file materials-16-03873-s001.zip › EBSD 10 mm distance from the center.jpg]

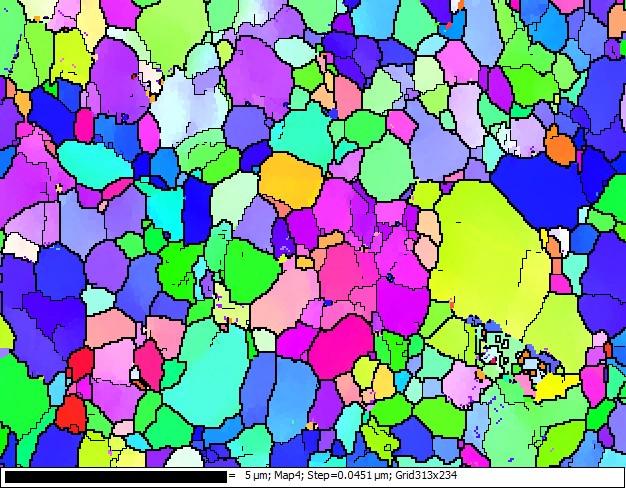

Supplement: Supplementary file 1 [file materials-16-03873-s001.zip › EBSD 2 mm distance from the center.jpg]

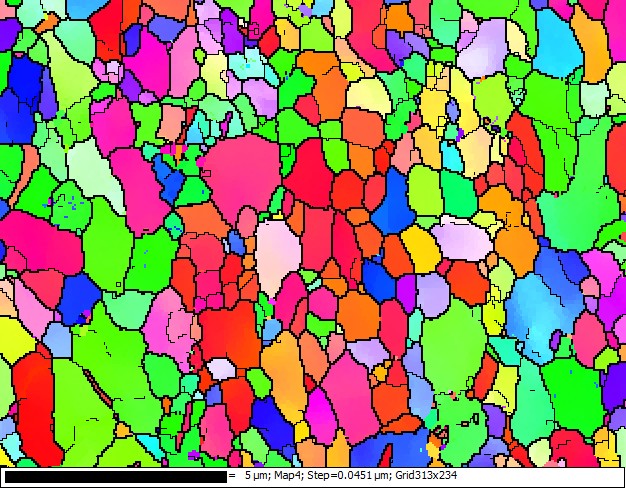

Supplement: Supplementary file 1 [file materials-16-03873-s001.zip › EBSD 4 mm distance from the center.jpg]

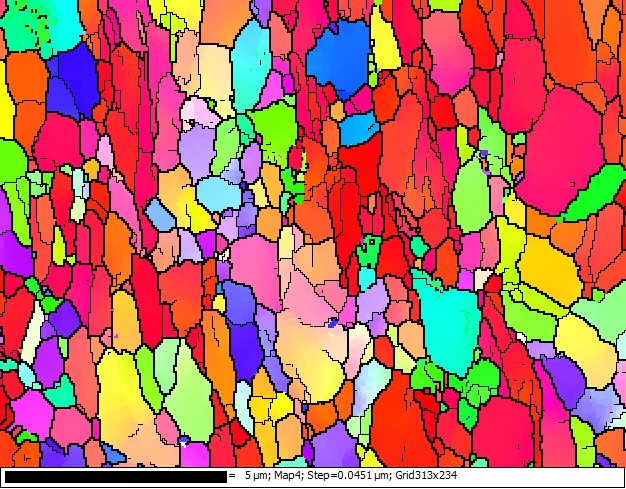

Supplement: Supplementary file 1 [file materials-16-03873-s001.zip › EBSD 6 mm distance from the center.jpg]

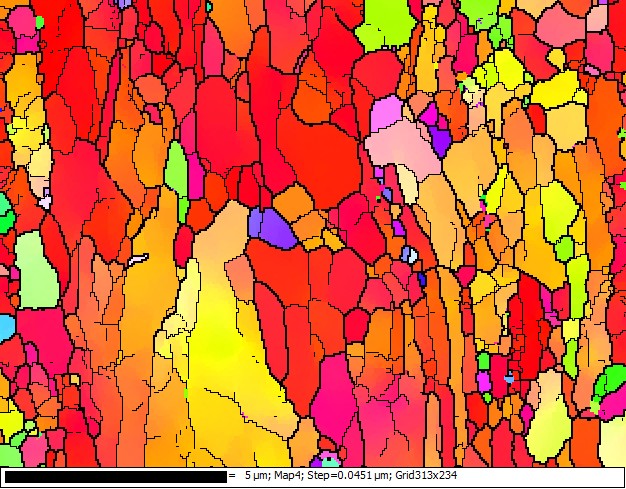

Supplement: Supplementary file 1 [file materials-16-03873-s001.zip › EBSD 8 mm distance from the center.jpg]

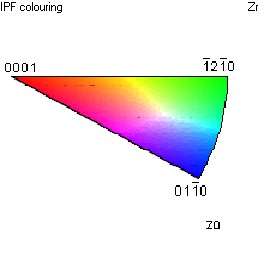

Supplement: Supplementary file 1 [file materials-16-03873-s001.zip › IPF_colouring.jpg]

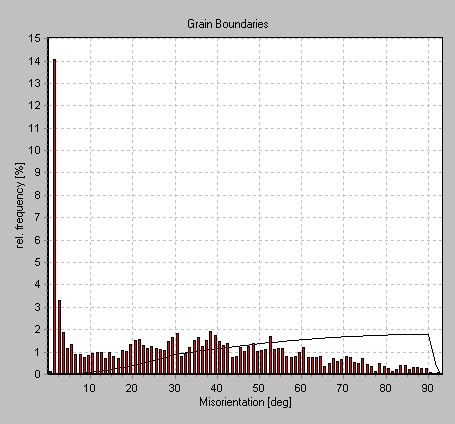

Supplement: Supplementary file 1 [file materials-16-03873-s001.zip › Misorientation 0 mm distance from the center.jpg]

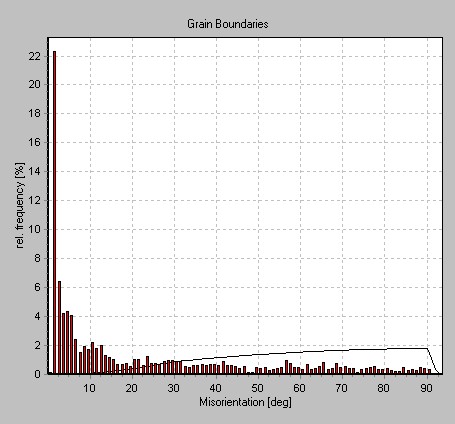

Supplement: Supplementary file 1 [file materials-16-03873-s001.zip › Misorientation 10 mm distance from the center.jpg]

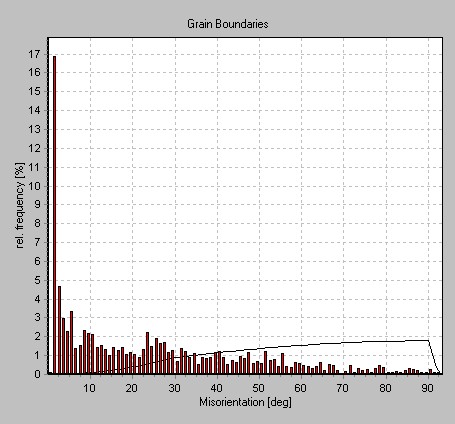

Supplement: Supplementary file 1 [file materials-16-03873-s001.zip › Misorientation 2 mm distance from the center.jpg]

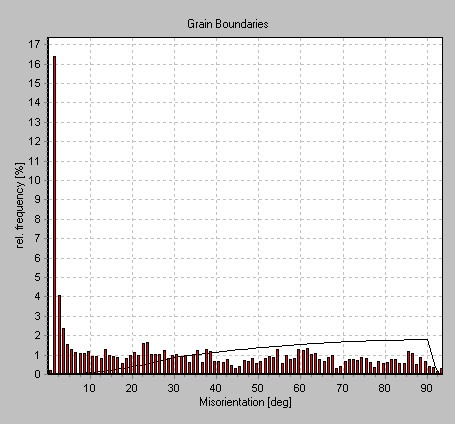

Supplement: Supplementary file 1 [file materials-16-03873-s001.zip › Misorientation 4 mm distance from the center.jpg]

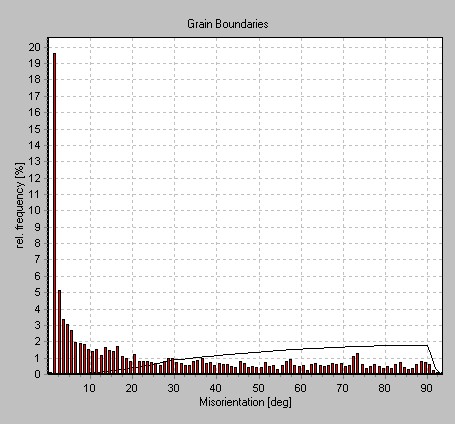

Supplement: Supplementary file 1 [file materials-16-03873-s001.zip › Misorientation 6 mm distance from the center.jpg]

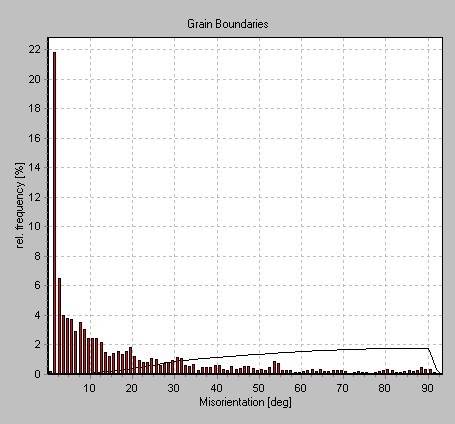

Supplement: Supplementary file 1 [file materials-16-03873-s001.zip › Misorientation 8 mm distance from the center.jpg]
